# When Cronbach’s Alpha Is Not Enough: Implications for the Clinical Use of Vision-Related Patient-Reported Outcomes

**DOI:** 10.3390/vision10030052

**Published:** 2026-08-06

**Authors:** Mario Cantó-Cerdán, María C. Martínez-Hergueta, Ana Siverio-Colomina, Pilar Yébana-Rubio, Jorge Alió-del-Barrio, Pilar Cacho-Martínez, Ángel García-Muñoz, Antonio Martínez-Abad

**Affiliations:** 1 Vissum (Miranza Group), 03016 Alicante, Spain; hergueta90@gmail.com (M.C.M.-H.); anaisabel.siverio@vissum.com (A.S.-C.); pilar.yebana@vissum.com (P.Y.-R.); jorge_alio@hotmail.com (J.A.-d.-B.); antonio.martinez@vissum.com (A.M.-A.); 2 Grupo de Investigación en Optometría (GIOptom), University of Alicante, 03690 Alicante, Spain; cacho@ua.es (P.C.-M.); ag.munoz@ua.es (Á.G.-M.); 3 Ophthalmology Department, Hospital General de Elda, 03600 Alicante, Spain; 4 Division of Ophthalmology, School of Medicine, Universidad Miguel Hernández, 03550 Alicante, Spain

**Keywords:** classical test theory, Rasch analysis, Cronbach’s alpha, person separation ratio, patient-reported outcomes, measurement reliability, vision

## Abstract

Patient-reported outcome measures (PROMs) in vision science are commonly evaluated using Cronbach’s alpha, yet Rasch-based statistics may provide different information about their reliability. This study compared Cronbach’s alpha, a Classical Test Theory (CTT) index, with Rasch person reliability and the person separation ratio across three vision-related questionnaires: the Symptom Questionnaire for Visual Dysfunctions (SQVD; *n* = 434), the Ocular Surface Disease Index (OSDI; *n* = 294), and the Quality of Vision questionnaire (QoV; *n* = 288), using retrospective data from unselected clinical populations. Cronbach’s alpha indicated acceptable internal consistency across all questionnaires (α = 0.80–0.87), whereas Rasch person reliability was lower (0.62–0.80) and person separation ratios ranged from 1.29 to 2.00, indicating more limited discrimination. Item reliability was generally high (0.90–0.97) overall but declined in subgroups with restricted score variability. Discrepancies were most pronounced in the OSDI, where estimates approached their theoretical floor when subgroups were defined by the questionnaire’s own scores. Cronbach’s alpha and Rasch-based statistics are not interchangeable and capture different aspects of measurement precision: alpha may overestimate internal consistency, whereas person reliability and the separation ratio provide complementary information about discriminatory capacity. These findings support reporting both CTT and Rasch-based reliability statistics when evaluating vision-related PROMs in clinical populations.

## 1. Introduction

Patient-reported outcomes (PROs) are widely used in vision research to quantify symptoms, visual functioning, and perceived quality of vision, providing information that cannot be obtained from objective clinical measures alone [1,2,3]. In ophthalmology and optometry, PRO questionnaires are commonly applied in clinical and epidemiological studies to evaluate visual dysfunctions, ocular surface conditions, and outcomes following refractive or intraocular surgery. Consequently, the measurement properties of these instruments are critical for ensuring the validity and interpretability of findings in vision research.

Reliability assessment of PRO questionnaires has traditionally relied on Classical Test Theory (CTT), with Cronbach’s alpha being the most frequently reported index of internal consistency [4]. Cronbach’s alpha estimates the extent to which item responses covary within a scale and therefore reflects the consistency with which the items measure a common construct. It is calculated from the number of items, their variances, and the covariance between item responses, with values closer to 1 indicating greater internal consistency. However, Cronbach’s alpha was originally developed under restrictive assumptions, including tau-equivalence of items and uncorrelated measurement errors [4,5]. These assumptions are rarely satisfied in vision-related PRO instruments, which typically include heterogeneous items and ordinal response categories. Furthermore, Cronbach’s alpha is influenced by the number of items, may be artificially inflated by item redundancy, and is calculated using ordinal raw scores often treated as interval-level data [6,7,8,9]. In epidemiological studies, where PRO scores are used to compare populations or assess associations with clinical variables, these limitations may compromise the interpretability of results.

Modern measurement approaches based on item response theory, particularly Rasch analysis, have gained increasing acceptance in vision science as a more robust alternative [1,10,11]. Rasch models transform ordinal responses into interval-level measures, enable detailed evaluation of item functioning, and support assessment of measurement invariance across subgroups. These properties are especially relevant in epidemiological and clinical research, where comparability across populations is essential. Within the Rasch framework, reliability is quantified using person reliability and the person separation ratio, which respectively reflect the reproducibility of person ordering and the ability of an instrument to distinguish between individuals with different levels of the latent trait on a linear scale [11]. Although Cronbach’s alpha and Rasch person reliability are mathematically related and share the same 0–1 scale, they are derived from different data structures, ordinal raw scores versus interval-level Rasch measures, and converge only under restrictive conditions uncommon in clinical and population-based vision studies [10,12].

Despite the increasing use of Rasch analysis in the development and validation of vision-related PRO instruments, Cronbach’s alpha continues to be routinely reported as the primary reliability index, even for questionnaires developed or validated within a Rasch framework [13,14]. This practice introduces a methodological inconsistency, as reliability is often summarized using a coefficient that is not aligned with the underlying measurement model. Several widely used instruments in vision science illustrate this issue, including the Quality of Vision questionnaire [15], the Symptom Questionnaire for Visual Dysfunctions [16,17], and the Ocular Surface Disease Index [18]. These three instruments were selected for the present comparison precisely because each has an existing Rasch-based validation, allowing for a direct, instrument-anchored evaluation of the concordance between Cronbach’s alpha and Rasch-derived reliability indices. Although these instruments have been developed or re-evaluated using Rasch analysis, Cronbach’s alpha is still frequently reported as the primary reliability estimate, while Rasch-based indices such as person reliability and the person separation ratio are often underutilized or omitted. This inconsistency may affect comparability across studies and complicate the interpretation of PRO-based outcomes. However, the extent to which Cronbach’s alpha and Rasch-based reliability estimates provide convergent or divergent information in real-world vision-related datasets remains insufficiently explored.

The aim of this study was to compare Cronbach’s alpha, Rasch person reliability, and the person separation ratio in three commonly used vision-related PROMs applied to distinct clinical populations. A secondary objective was to evaluate the extent to which these statistics provide convergent or divergent information about measurement performance across overall samples and clinically defined subgroups.

## 2. Materials and Methods

### 2.1. Study Design and Sample Selection

This study followed a retrospective observational design based on the secondary analysis of existing datasets obtained during the development and validation of three vision-related PRO questionnaires. Independent samples were analysed for each questionnaire in order to preserve their original clinical context and intended application. All three datasets comprised consecutive patients attending routine clinical practice in Spain, rather than preselected or artificially enriched research cohorts, and were collected between 2019 and 2025. Questionnaires were administered in Spanish (Castilian) and were completed under the guidance of clinical staff in all cases.

The final sample consisted of 434 participants for the Symptom Questionnaire for Visual Dysfunctions (SQVD), 288 for the Quality of Vision questionnaire (QoV), and 294 for the Ocular Surface Disease Index (OSDI). Subgroup sample sizes are detailed within each corresponding analysis.

For the SQVD dataset, participants were patients attending optometric examinations who presented with refractive, accommodative, and/or binocular dysfunctions. For the QoV dataset, the sample included patients who had undergone intraocular lens implantation (cataract surgery or refractive lensectomy) and completed the questionnaire during the far postoperative period, six months after surgery. For the OSDI dataset, participants were individuals attending routine eye examinations, including subjects both with and without dry eye symptoms.

Although these datasets correspond to different clinical populations, their comparison is methodologically justified as the objective of this study is to evaluate differences between reliability indices rather than between clinical conditions.

Only anonymized data were used in the present analyses. Ethical approval had been obtained for the original data collection procedures, and all studies adhered to the principles of the Declaration of Helsinki.

### 2.2. Inclusion and Exclusion Criteria

For the SQVD dataset, inclusion criteria comprised patients who had completed a comprehensive optometric examination and were diagnosed with at least one refractive, accommodative, or binocular dysfunction. Subjects with ocular pathology unrelated to visual dysfunctions, previous ocular surgery, or systemic conditions affecting visual function were excluded.

For the QoV dataset, inclusion criteria included patients who had undergone intraocular lens implantation and completed the questionnaire at six months post-operation (far postoperative period). Subjects with postoperative complications, concurrent ocular pathology unrelated to the surgical procedure, or incomplete questionnaire responses were excluded.

For the OSDI dataset, inclusion criteria included adults attending routine eye examinations who completed the questionnaire regardless of dry eye status. Participants classified as no dry eye were not deliberately recruited asymptomatic controls, but consecutive patients attending routine eye examinations for reasons unrelated to dry eye who did not meet the symptom threshold described below. Subjects with active ocular infection, recent ocular surgery, or incomplete questionnaire data were excluded.

Across all datasets, only participants with complete questionnaire responses were included to ensure comparability between reliability indices. No imputation procedures were applied. No participants were excluded due to missing responses, as questionnaires were administered under clinical staff supervision and only fully completed questionnaires were recorded in the clinical database.

### 2.3. Questionnaires and Scoring

Three vision-related PRO questionnaires were analysed: the SQVD, the QoV questionnaire, and the OSDI. Each instrument assesses a distinct visual construct and uses ordinal response categories.

The SQVD [17] consists of 14 items assessing the presence and frequency of visual symptoms associated with refractive, accommodative, and binocular dysfunctions. Each item is scored on a three-category ordinal scale (“No” = 0, “Occasionally/Often” = 1, “Almost always” = 2), yielding a total score ranging from 0 to 28, with higher scores indicating greater symptomatology.

The QoV [15] questionnaire evaluates subjective quality of vision across three subscales: frequency, severity, and bothersome. It includes 10 visual symptoms assessed across these three domains (30 items in total). Each item is scored on a four-category ordinal scale (0–3). Raw scores are calculated independently for each subscale and transformed to a 0–100 scale, with higher scores indicating poorer perceived quality of vision.

The OSDI [18] is a 12-item questionnaire designed to assess symptoms related to ocular surface disease. Items are scored on a five-category ordinal scale ranging from 0 to 4. The total score is calculated using a standardized formula and expressed on a 0–100 scale, with higher scores indicating greater symptom severity.

### 2.4. Statistical Analysis and Psychometric Analysis

Psychometric analyses were conducted separately for each questionnaire to preserve their conceptual and clinical specificity. The primary objective was to compare reliability estimates derived from CTT and Rasch measurement theory.

Cronbach’s alpha was calculated from the raw ordinal responses to the items contributing to each scale. For the SQVD and OSDI, alpha was calculated across all items contributing to the total questionnaire score. For the QoV questionnaire, alpha was calculated separately for the frequency, severity, and bothersome subscales. Cronbach’s alpha reflects the internal consistency of the item responses, with values ≥ 0.70 considered acceptable for group-level comparisons [4].

**Figure 1:**
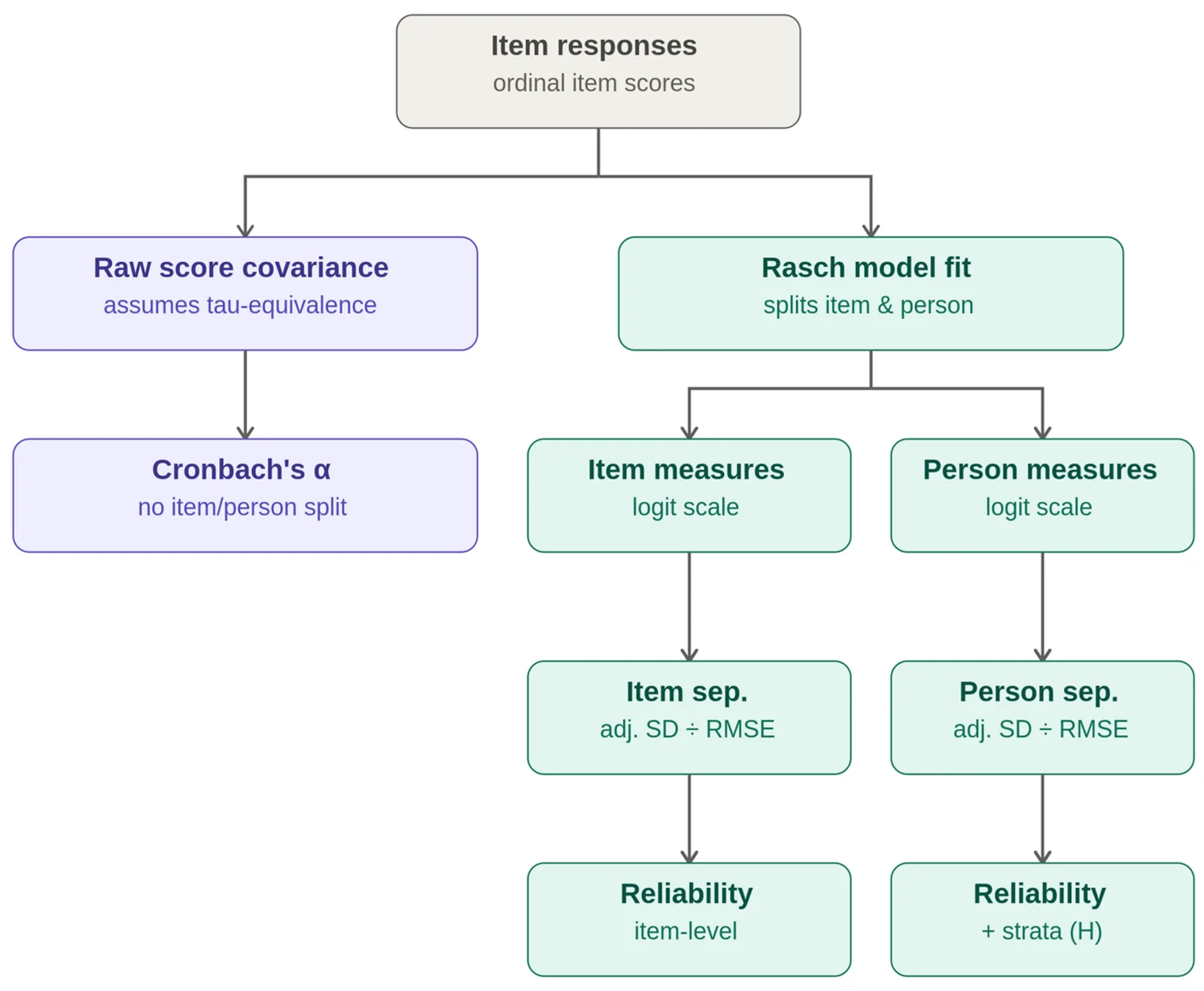
Conceptual comparison between Classical Test Theory and Rasch measurement approaches to reliability. Cronbach’s alpha is calculated from the covariance among raw ordinal item scores under the assumption of tau-equivalence, yielding a single reliability coefficient that does not distinguish between item- and person-level measurement precision. In contrast, Rasch analysis estimates item and person measures separately on a common interval (logit) scale, from which independent item and person separation ratios (adjusted standard deviation ÷ root mean square measurement error) are derived. Each separation ratio can be transformed into a reliability coefficient (R = G^2^/(1 + G^2^)) and, for person measures, into the number of statistically distinguishable strata (H = (4G + 1)/3).

Rasch analyses were conducted separately for each questionnaire using polytomous Rasch models. The SQVD was analysed using the Andrich Rating Scale Model, which assumes a common category-threshold structure across items. The OSDI was analysed using the Andrich Rating Scale Model, and each QoV subscale was analysed using the Andrich Rating Scale Model. The selected models were consistent with the response-category structure and the analytical specifications applied to each questionnaire. Person and item measures were estimated on a logit scale using Winsteps version 4.8.1. Rasch-based reliability was evaluated using person reliability, item reliability, and their corresponding separation ratios, as reported by Winsteps. Person reliability reflects the reproducibility of the ordering of participants according to their estimated level of the latent trait. Item reliability reflects the reproducibility of the item-difficulty hierarchy across comparable samples. Both reliability coefficients range from 0 to 1. The person separation ratio represents the adjusted standard deviation of person measures relative to their root mean square measurement error and indicates the extent to which the instrument differentiates between participants. Similarly, the item separation ratio represents the spread of item difficulties relative to their measurement error and indicates the precision with which the item hierarchy has been established. Separation ratios are not restricted to the 0–1 interval. Reliability and separation are related according to R = G^2^/(1 + G^2^), where R denotes reliability and G denotes the corresponding separation ratio. Figure 1 summarizes this distinction: Cronbach’s alpha yields a single undifferentiated coefficient for the whole set of items and persons, whereas the Rasch model separately calibrates item and person measures, from which independent item and person reliability statistics, and, for persons, the number of statistically distinguishable strata, H = (4G + 1)/3, can be derived. A person separation ratio ≥ 2.0, corresponding to a person reliability of approximately 0.80, was considered indicative of adequate person discrimination for group-level analyses. An item separation ratio ≥ 3.0, corresponding to an item reliability of approximately 0.90, was considered indicative of a sufficiently reproducible item-difficulty hierarchy [13,16].

Subgroup analyses were performed to explore differences in reliability across clinical contexts. For the SQVD, participants were stratified into those with diagnosed visual dysfunctions and those without dysfunction, based on findings from a comprehensive optometric examination (assessment of refractive, accommodative, and binocular function) that was independent of the SQVD score itself. For the OSDI dataset, participants were classified according to symptom severity based on standard thresholds (≤12: no dry eye; ≥13: dry eye) [18]. This classification was based on questionnaire scores and may introduce range restriction; because reliability statistics are variance-dependent, this can mechanically reduce reliability estimates within subgroups regardless of the instrument’s discriminative performance in an unselected sample. For the QoV questionnaire, participants were stratified according to intraocular lens type (multifocal vs. extended depth-of-focus). Cronbach’s alpha, Rasch person reliability, person and item separation ratios, targeting, and the first contrast eigenvalue were calculated independently for each subgroup.

To facilitate comparison between classical and Rasch-based reliability estimates, Cronbach’s alpha and Rasch person reliability were reported side by side because both are expressed as coefficients ranging from 0 to 1. The person separation ratio was reported as an additional Rasch-based indicator of the extent to which the instrument discriminated between participants with different levels of the latent trait. Differences between the estimates were examined descriptively, focusing on their magnitude, direction, and relationship with targeting and score variability. Differences in questionnaire scores between subgroups were analyzed using the Mann–Whitney U test, given the ordinal nature and potential non-normal distribution of the data. A significance level of *p* < 0.05 was adopted.

Statistical analyses based on Classical Test Theory were performed using SPSS version 26.0 (IBM Corp., Armonk, NY, USA). Rasch analyses were conducted using Winsteps version 4.8.1 (Winsteps.com, Chicago, IL, USA).

## 3. Results

### 3.1. Sample Characteristics

A total of 1016 participants were included in the study. The SQVD dataset comprised 434 participants, the OSDI dataset 294 participants, and the QoV dataset 288 participants. The overall mean age was 49.43 ± 19.13 years (range: 8–87), and 53.4% of participants were female (Table 1).

### 3.2. Questionnaire Scores

Descriptive statistics for questionnaire scores are presented in Table 2.

For the SQVD, the mean total score was 6.18 ± 4.62. Participants with visual dysfunction showed significantly higher scores than those without dysfunction (*p* < 0.001).

For the OSDI, the mean score was 11.11 ± 9.06. Participants classified as having dry eye symptoms presented higher scores than those without dry eye (*p* < 0.001).

For the QoV questionnaire, mean scores were 30.30 ± 18.69 for frequency, 25.63 ± 16.56 for severity, and 22.64 ± 18.32 for bothersome. No statistically significant differences were found between intraocular lens groups (EDOF vs. multifocal) for any of the three subscales (*p* > 0.05).

### 3.3. Internal Consistency and Rasch-Based Reliability

Reliability estimates are summarized in Table 3.

Cronbach’s alpha values were within acceptable ranges for all questionnaires in the overall sample, with values of 0.81 for the SQVD, 0.87 for the OSDI, and between 0.80 and 0.85 across the QoV subscales.

Rasch-based reliability differed across instruments. In the overall samples, person reliability was approximately 0.80 for the SQVD, 0.79 for the OSDI, and between 0.62 and 0.66 across the QoV subscales. The corresponding person separation ratios were 2.00 for the SQVD, 1.96 for the OSDI, and between 1.29 and 1.39 for the QoV subscales. Thus, adequate person separation was observed for the SQVD, while the OSDI was marginally below the predefined threshold and the QoV subscales showed more limited discrimination.

### 3.4. Rasch Model Diagnostics

Rasch model parameters are presented in Table 3. Item reliability was high across most overall samples, ranging from 0.90 in the SQVD to 0.97 in the QoV frequency and severity subscales, with corresponding item separation ratios between 3.05 and 6.14, generally meeting the predefined threshold for a reproducible item-difficulty hierarchy.

Targeting values were negative across all questionnaires and subgroups, ranging from −1.48 logits in the SQVD overall sample to −3.16 logits in the OSDI no dry eye subgroup.

Unidimensionality analysis based on principal component analysis of residuals showed first contrast eigenvalues below 2.0 for most datasets. Values slightly above this threshold were observed in the OSDI overall sample (2.03), the dry eye subgroup (2.65), and in some QoV subgroups.

### 3.5. Subgroup Analyses

Subgroup analyses revealed variations in reliability estimates across clinical groups.

In the SQVD dataset, person separation ratios were 2.05 in participants with visual dysfunction and 1.94 in those without dysfunction. Cronbach’s alpha values showed minor variation between subgroups. Targeting was −1.08 logits in the dysfunction group and −2.21 logits in the non-dysfunction group. Item reliability decreased from 0.89 in the dysfunction group to 0.78 in the non-dysfunction group, with item separation ratios of 2.79 and 1.86, respectively, the latter falling below the predefined threshold.

In the OSDI dataset, both Cronbach’s alpha and the person separation ratio decreased in subgroup analyses. Cronbach’s alpha was 0.47 in the dry eye group and 0.16 in the no dry eye group, while person separation ratios were 0.98 and 0.00, respectively. Targeting values were −0.71 logits in the dry eye group and −3.16 logits in the no dry eye group. Item reliability was 0.89 in the dry eye group and 0.85 in the no dry eye group, with item separation ratios of 2.80 and 2.42, respectively, both below the predefined threshold. Both estimates approached their theoretical floor in the no dry eye subgroup, coinciding with the reduced score variability introduced by this classification criterion.

In the QoV dataset, Cronbach’s alpha values were comparable between EDOF and multifocal groups across all subscales. Person separation values showed moderate variation between groups, with slightly higher values observed in the EDOF group across subscales. Item reliability and item separation ratios were comparable between EDOF and multifocal groups across all subscales (0.91–0.95 and 3.17–4.46, respectively), indicating similarly reproducible item hierarchies in both groups.

## 4. Discussion

The present study offers an empirical comparison of reliability estimates derived from Classical Test Theory and Rasch measurement theory across three commonly used vision-related questionnaires, applied to unselected clinical samples. The main findings indicate that, although all instruments demonstrated acceptable levels of internal consistency when assessed using Cronbach’s alpha, Rasch-based metrics revealed important limitations in their measurement performance. In particular, the person separation ratio consistently indicated a lower ability of the questionnaires to discriminate between individuals, especially in specific subgroups. These results suggest that traditional interpretations of internal consistency may not adequately reflect the true measurement properties of patient-reported outcome measures.

The analysis of Table 3 further highlights a consistent discrepancy between both approaches. While Cronbach’s alpha values were generally within ranges considered acceptable or high across all instruments, person separation ratios were lower and more variable, particularly in subgroup analyses. These findings confirm that both statistics are not interchangeable and capture fundamentally different aspects of measurement. Cronbach’s alpha primarily reflects inter-item correlation and internal consistency, whereas the person separation ratio reflects the ability of an instrument to discriminate between individuals across levels of the latent trait. This divergence suggests that reliance on Cronbach’s alpha alone may lead to an overestimation of measurement quality.

In the SQVD, the overall analysis showed acceptable internal consistency (α = 0.81), while the person separation ratio was 2.00, indicating borderline adequate discrimination. In subgroup analyses, the person separation ratio increased slightly in participants with visual dysfunction (2.05) and decreased in those without dysfunction (1.94), while Cronbach’s alpha showed only minor variation. This pattern reflects the different dependencies of both metrics. Cronbach’s alpha is influenced by inter-item correlation and may increase when items behave similarly or redundantly [19,20]. In contrast, the person separation ratio depends on the spread of person measures relative to item difficulty. In symptomatic individuals, a wider distribution of the latent trait enhances discrimination, whereas in asymptomatic subjects, restricted variability leads to reduced discrimination. This effect is consistent with the observed targeting differences (−1.08 vs. −2.21 logits) and aligns with previous findings highlighting targeting limitations in low-symptom populations [16]. These results illustrate that high internal consistency does not necessarily imply adequate measurement performance.

For the OSDI, the overall analysis revealed high internal consistency (α = 0.87), while the person separation ratio was 1.96, below the recommended threshold for adequate discrimination. This discrepancy became more pronounced in subgroup analyses, where both Cronbach’s alpha and the person separation ratio decreased substantially (α = 0.47 and 0.16; person separation ratio = 0.98 and 0.00 for dry eye and no dry eye groups, respectively). A plausible explanation lies in the classification approach used, as subgroup definition was based on OSDI scores themselves. This introduces circularity and restricts score variability, reducing both inter-item covariance and discriminatory capacity. This range restriction is an expected mathematical consequence of any variance-based reliability statistic when subgroups are defined from the same score being evaluated, rather than evidence that the OSDI performs poorly as a measurement instrument in an unselected clinical population. This interpretation is further supported by the pattern observed in the SQVD and QoV questionnaires, where subgroups were defined using criteria external to the instrument’s own score (clinical diagnosis of visual dysfunction and intraocular lens type, respectively): in both cases, Cronbach’s alpha and the person separation ratio showed only minor to moderate variation between subgroups, in clear contrast with the marked decline of both indices observed in the OSDI when subgroups were defined by the questionnaire’s own score. This contrast reinforces that the deterioration observed in the OSDI subgroups reflects the circularity of the classification criterion rather than a generalizable limitation of subgroup analysis itself. The overall sample estimates (person reliability = 0.79; separation ratio = 1.96), which are not affected by this circularity, are therefore more informative about the instrument’s genuine discriminative capacity than the subgroup-level estimates. It should also be noted that the no dry eye subgroup was not composed of deliberately recruited healthy controls, but of unselected patients attending routine eye examinations for reasons unrelated to dry eye; the floor effect observed in this subgroup therefore reflects a realistic clinical screening scenario rather than the application of the instrument outside its intended population. These findings suggest that caution is warranted when reporting reliability statistics after stratifying a sample by the same score being evaluated, as such subgroup-level estimates may not reflect the instrument’s genuine performance in unselected clinical populations. Previous studies have emphasized that PROMs should complement, rather than replace, clinical assessment [1,2].

In the QoV questionnaire, all subscales demonstrated high internal consistency (α = 0.80–0.85), whereas person separation ratios were lower (1.29–1.39), indicating limited discrimination. No significant differences were observed between multifocal and EDOF groups at the overall level. However, subgroup analyses showed a consistent pattern in which Cronbach’s alpha increased while the person separation ratio decreased in the EDOF group, characterized by lower symptom scores. This pattern reflects reduced variability and increased response homogeneity. When responses cluster at the lower end of the scale, inter-item correlations increase, inflating Cronbach’s alpha, while the reduced spread of person measures limits discrimination. From a Rasch perspective, this corresponds to suboptimal targeting, where item difficulty does not align with the distribution of person abilities [13,21]. These findings further illustrate that high internal consistency may coexist with limited measurement sensitivity.

Item reliability and item separation provided information complementary to person reliability. Whereas person reliability reflects the reproducibility of the ordering of participants, item reliability reflects the stability of the item-difficulty hierarchy. Item reliability was high in most overall analyses, indicating that the available sample sizes were generally sufficient to locate the items reproducibly along the latent trait. Lower item separation in some subgroup analyses should not be interpreted as evidence of poorer internal consistency or inadequate person discrimination. Rather, it indicates reduced precision in confirming the relative hierarchy of item difficulties within those smaller or more restricted samples.

These discrepancies carry direct implications for clinical decision-making, particularly when these questionnaires are used to monitor patients over time or to compare individuals. Expressing the person separation ratio as the number of statistically distinguishable strata (H = (4G + 1)/3) [21] makes this concrete: in the OSDI, the overall H of approximately 3 strata falls to below 1 in the no dry eye subgroup, indicating that small score changes in low-symptom patients being monitored for dry eye should not be interpreted as clinically meaningful improvement or worsening, despite the questionnaire’s high overall alpha. In the QoV, an H of approximately 2 strata across subscales suggests that, when counseling patients after intraocular lens implantation, the instrument may reliably distinguish only between broadly low and high perceived visual disturbance rather than finer gradations of visual quality. In the SQVD, although person-level discrimination remained stable across subgroups, item reliability declined toward the low-symptom end of the scale (item separation ratio falling from 2.79 to 1.86 in the dysfunction and no-dysfunction subgroups, respectively); in the context of monitoring vision therapy or an optical correction, this indicates that total scores can reliably track overall symptomatic improvement, but the specific order in which individual symptoms are expected to resolve becomes less reproducible as patients approach the asymptomatic range. None of these clinically relevant nuances would have been apparent from Cronbach’s alpha alone.

Some limitations of this study should be acknowledged. First, the retrospective nature of data collection may have introduced selection bias, as participants were drawn from routine clinical practice rather than a prospectively defined sampling frame. Because reliability and separation indices are inherently dependent on sample variance, as demonstrated throughout this study, differences in case-mix, referral patterns, or symptom severity between retrospective and prospectively recruited cohorts could shift these estimates independently of the instruments’ true measurement properties. Sample size and subgroup distribution may further influence the stability of Rasch estimates, particularly in analyses with restricted variability. Second, the use of questionnaire-based criteria for subgroup classification, particularly in the OSDI dataset, introduces circularity and may affect the interpretation of psychometric results. Third, differences in questionnaire structure and dimensionality may limit direct comparability across instruments. Fourth, detailed clinical variables such as visual acuity, refractive error, contact lens wear, or objective ocular surface findings (e.g., tear break-up time, corneal staining) were not available for these retrospective datasets. The absence of such externally defined clinical variables, particularly for the OSDI dataset, precluded an additional non-circular subgroup stratification and limits the extent to which the observed differences in reliability estimates can be attributed to the measurement properties of each instrument rather than to the underlying clinical composition of each sample; this represents a relevant direction for future prospective research. Fifth, all datasets were collected using the Spanish versions of the SQVD, OSDI, and QoV administered to Spanish clinical populations. Because both Cronbach’s alpha and Rasch-based reliability indices are sample- and context-dependent, as shown throughout this study, the present findings should not be assumed to generalize directly to other language adaptations or to populations with different symptom prevalence, healthcare-seeking behavior, or cultural response patterns. Replication of this comparison using translated versions of these questionnaires and in other populations is warranted before extending these conclusions beyond the Spanish clinical context.

Despite these limitations, this study has several strengths. It offers an empirical illustration of how Classical Test Theory and Rasch-based reliability estimates can diverge across multiple vision-related PROMs, using data from unselected, consecutively recruited clinical populations rather than idealized or preselected samples, an approach that remains relatively uncommon. The inclusion of subgroup analyses offers valuable insight into how measurement properties behave across different clinical contexts, while also illustrating how the clinical composition of a sample can influence reliability estimates independently of instrument quality. Furthermore, the use of real clinical data enhances the applicability of the findings to both research and clinical practice.

## 5. Conclusions

In conclusion, the findings of this study indicate that Cronbach’s alpha, Rasch person reliability, and the person separation ratio provide complementary but fundamentally different information about the reliability of vision-related questionnaires. While Cronbach’s alpha consistently suggests acceptable internal consistency, Rasch-based statistics reveal important limitations in the ability of these instruments to discriminate between individuals. Given that Cronbach’s alpha is influenced by item characteristics and sample variability, it may overestimate measurement quality when used in isolation. In contrast, Rasch-based statistics offer a more informative assessment of discriminatory capacity within the Rasch framework. From a practical perspective, we recommend reporting person reliability alongside the person separation ratio rather than relying on either statistic alone: person reliability allows for direct comparison with Cronbach’s alpha on the same 0–1 scale, whereas the separation ratio conveys the instrument’s discriminative range without the ceiling imposed by a bounded coefficient. These considerations are not limited to vision science and may be extended to other areas of health research where patient-reported outcomes are used. Clinicians should consider complementing Cronbach’s alpha with Rasch-based statistics such as person reliability and the person separation ratio when interpreting PROMs in clinical optometric settings. Future validation studies of vision-related PROMs should routinely report both Classical Test Theory and Rasch-based reliability indices to enable a more complete appraisal of measurement quality.

## Figures and Tables

**Table 1:** Demographic characteristics of the study sample by questionnaire. Data are presented as number of participants (N), mean age ± standard deviation (SD), age range (years), and sex distribution (n, %). SQVD, Symptom Questionnaire for Visual Dysfunctions; OSDI, Ocular Surface Disease Index; QoV, Quality of Vision questionnaire; SD, standard deviation.

Questionnaire	N	Age (Mean ± SD)	Range	Female (%)	Male (%)
**SQVD**	434	35.44 ± 16.97	8–87	280 (64.5)	154 (35.5)
**OSDI**	294	54.16 ± 14.22	18–81	105 (35.7)	189 (64.3)
**QoV**	288	65.69 ± 8.30	45–82	158 (54.9)	130 (45.1)

**Table 2:** Descriptive statistics of questionnaire scores overall and by subgroup. Data are presented as number of participants (n, %), mean score, standard deviation (SD), and observed range. *p*-values indicate comparisons between subgroups within each questionnaire. SQVD, Symptom Questionnaire for Visual Dysfunctions; OSDI, Ocular Surface Disease Index; QoV, Quality of Vision questionnaire; EDOF, extended depth-of-focus intraocular lens; SD, standard deviation.

Questionnaire	Group	n (%)	Mean	SD	Range	*p*-Value (Between Groups)
**SQVD**						
	Overall	434 (100)	6.18	4.62	0–21	
	Dysfunction	248 (57.1)	8.32	4.41	0–21	<0.001
	No dysfunction	186 (42.9)	3.55	3.28	0–19	
**OSDI**						
	Overall	294 (100)	11.11	9.06	0–38	
	Dry eye	120 (40.8)	20.99	4.88	13–38	<0.001
	No dry eye	174 (59.2)	4.30	2.89	0–10	
**QoV**						
Frequency	Overall	288 (100)	30.30	18.69	0–85.41	
	EDOF	156 (54.2)	29.39	20.18	0–85.41	0.260
	Multifocal	132 (45.8)	31.38	16.74	0–70.73	
Severity	Overall	288 (100)	25.63	16.56	0–80.12	
	EDOF	156 (54.2)	25.48	17.84	0–80.12	0.761
	Multifocal	132 (45.8)	25.80	14.95	0–65.53	
Bothersome	Overall	288 (100)	22.64	18.32	0–83.37	
	EDOF	156 (54.2)	21.84	18.95	0–83.37	0.244
	Multifocal	132 (45.8)	23.59	17.57	0–69.39	

**Table 3:** Classical and Rasch-based reliability statistics and Rasch model parameters for each questionnaire and subgroup. Cronbach’s alpha was calculated from raw ordinal item responses. Rasch-based statistics include person reliability, the person and item separation ratios, targeting expressed as the mean person-item difference in logits, and the first contrast eigenvalue obtained from principal component analysis of Rasch residuals. SQVD, Symptom Questionnaire for Visual Dysfunctions; OSDI, Ocular Surface Disease Index; QoV, Quality of Vision questionnaire; EDOF, extended depth-of-focus intraocular lens.

Questionnaire	Group	Cronbach’s Alpha	Rasch Person Reliability	Person Separation Ratio	Rasch Item Reliability	Item Separation Ratio	Targeting	First Contrast Eigenvalue
**SQVD**								
	Overall	***0.81***	***0.80***	***2.00***	***0.90***	***3.05***	−1.48	***1.6921***
	Dysfunction	***0.74***	***0.81***	***2.09***	0.89	2.79	−1.08	***1.7521***
	No dysfunction	***0.75***	0.79	1.94	0.78	1.86	−2.21	***1.8493***
**OSDI**								
	Overall	***0.87***	0.79	1.96	***0.92***	***3.43***	−2.21	2.0262
	Dry eye	0.47	0.49	0.98	0.89	2.80	−0.71	2.6473
	No dry eye	0.16	0.00	0.00	0.85	2.42	−3.16	***1.6529***
**QoV**								
Frequency	Overall	***0.80***	0.62	1.29	***0.97***	***6.14***	−1.56	***1.8689***
	EDOF	***0.84***	0.68	1.46	***0.95***	***4.35***	−1.89	***1.8614***
	Multifocal	***0.77***	0.59	1.19	***0.95***	***4.46***	−1.36	2.0765
Severity	Overall	***0.84***	0.66	1.39	***0.97***	***5.70***	−1.63	***1.8557***
	EDOF	***0.87***	0.71	1.57	***0.94***	***3.97***	−1.67	***1.8166***
	Multifocal	***0.79***	0.61	1.24	***0.95***	***4.16***	−1.63	2.1780
Bothersome	Overall	***0.85***	0.64	1.33	***0.96***	***4.70***	−1.87	***1.8249***
	EDOF	***0.87***	0.67	1.43	***0.91***	***3.17***	−2.16	***1.7927***
	Multifocal	***0.83***	0.62	1.28	***0.93***	***3.57***	−1.68	2.1641

## Data Availability

The original contributions presented in this study are included in the article. Further inquiries can be directed to the corresponding author.

## Abbreviations

The following abbreviations are used in this manuscript:

CTT	Classical Test Theory
PROMs	Patient-reported outcome measures
PROs	Patient-reported outcomes
SQVD	Symptom Questionnaire for Visual Dysfunctions
OSDI	Ocular Surface Disease Index
QoV	Quality of Vision questionnaire
EDOF	Extended depth-of-focus (intraocular lens)
SD	Standard deviation
